# Clinical analysis of hyperbaric oxygen combined with subdural drilling and drainage in the management of subdural effusion type IV with intracranial infection in infant patients

**DOI:** 10.3389/fneur.2024.1340650

**Published:** 2024-02-26

**Authors:** Liuyin Chen, Yanke Yue, Pengyuan Luo, Yi Qu, Jiangshun Fang, Chaojun Xin, Lige Lv, Jimei Luan, Zhenghai Cheng, Zhiguo Yang, Yaning Sun

**Affiliations:** Department of Neurosurgery, Hebei Provincial Children’s Hospital, Shijiazhuang, Hebei, China

**Keywords:** infants, hyperbaric oxygen, subdural effusion, subdural drilling and drainage, intracranial infection

## Abstract

**Background:**

To explore the therapeutic effect of hyperbaric oxygen combined with subdural drilling and drainage (SDD) on subdural effusion type IV with intracranial infection in infant patients.

**Methods:**

This retrospective controlled study included 328 infant patients with subdural effusion type 4 with intracranial infection between January 2005 and January 2023. 178 patients were treated by hyperbaric oxygen combined with SDD (group A). 142 cases were treated with SDD (group B). 97 infants were only received hyperbaric oxygen (group C). Clinical outcomes, the control time of intracranial infection, complications, and the degree of brain re-expansion after 6 months of treatment were compared among the three groups. According to the comprehensive evaluation of treatment effectiveness and imaging results, it is divided into four levels: cured, significantly effective, improved, and ineffective.

**Results:**

No patient died during follow-up. The three groups were similar regarding age, sex, the general information, and clinical symptoms (*p* > 0.05). All intracranial infections in the children were effectively controlled. There was no difference in infection control time between group A and group B, and there was no statistical significance. However, the control time of intracranial infection between the two groups was different from that of group C, which was statistically significant. Compared with group B and group C, the degree of brain re-expansion in group A has obvious advantages and significant differences. The effective rates of the three groups were 83.7%, 58.5%, and 56.7%, respectively. There were 28 cases of subcutaneous hydrops in group A and 22 cases of subcutaneous hydrops in group B after operation, and no other serious complications.

**Conclusion:**

The SDD is safe and effective for infant patients with intracranial infections through fluid replacement and intrathecal antibacterial. Hyperbaric oxygen is effective as an adjuvant therapy to promote brain re-expansion.

## Introduction

Subdural effusion (SE) is a common disease, which can also be said to be a common complication. Common causes include trauma (1) and intracranial infection (2). And even a lot of unexplained SE. The child’s development is lagging behind, such as not being able to lift his head and roll over, and even language development is delayed. After visiting the hospital for examination, a CT scan of the head revealed SE. Most patients with SE do not require special treatment (3). However, a large amount of SE will compress the brain tissue, leading to brain tissue atrophy. Severe SE will lead to brain tissue softening and hydrocephalus, which will affect the development of nervous system of infants and seriously affect their behavior and cognition (4).

Intracranial infection is a very common disease in pediatrics. Although treatment methods are constantly being updated, some children still have neurological complications and sequelae (2, 5). SE is a common complication of intracranial infection, with an incidence rate of up to 50 to 70% (6). SE after intracranial infection is common in children under 1 year old, occurring mostly during the first to third weeks of intracranial infection (7). After the formation of SE, a large amount of fibrin will gradually deposit on the brain surface, forming an encapsulating cystic wall, which restricts the development of brain tissue. It is worth noting that SE under infection is difficult to absorb, and the brain tissue is chronically compressed, leading to permanent neurological dysfunction in some children (6). Therefore, early and correct treatment can effectively reduce the incidence of neurological sequelae and have a very positive effect on improving the prognosis of children.

Hyperbaric oxygen has been widely used in the treatment of children’s brain hypoplasia, hypoxic–ischemic encephalopathy and brain injury recovery (8). As an auxiliary treatment method, it can rapidly improve the hypoxia of brain tissue in the focus area, promote the diffusion of oxygen content in brain tissue, and promote the metabolism and proliferation of nerve cells, thereby facilitating the shallowing of cerebral sulci and the reduction of subdural space (9). Hyperbaric oxygen therapy has significant effects on the treatment of mild SE. However, the effectiveness of hyperbaric oxygen therapy in children with severe SE and intracranial infection still needs further research.

According to the results of cranial CT, the maximum thickness of subdural residual cavity (T Thickness) is expressed as IV types. Type I: T < 0.5 cm. Type II: 0.5 cm ≤ T < 1 cm. Type III: 1 cm ≤ T < 1.5 cm. Type IV T ≥ 1.5 cm. This study suggests that all SE with clinical symptoms above 1.5 cm should be treated with intervention therapy. Anterior fontanel puncture (10), SDD (2), subdural-peritoneal shunt (11) and even craniotomy (12) are all effective ways to treat SE. At present, there is no optimal treatment for SE with intracranial infection, and there is no sufficient evidence to indicate that one treatment is completely superior to another. To investigate the clinical efficacy of different treatment methods for SE with intracranial infection, this study analyzed 328 children with SE thickness ≥ 1.5 cm (type IV) accompanied by intracranial infection admitted to our hospital from January 2005 to January 2023. Under the premise of conventional medication, compare the effectiveness and safety of hyperbaric oxygen therapy, SDD, and hyperbaric oxygen combined with SDD in controlling intracranial infection and promoting brain re-expansion.

## Materials and methods

We reviewed 328 consecutive infant patients who underwent the different treatment methods for SE type IV with intracranial infection at Children’s Hospital of Hebei Province from January 2005 to January 2023. The institutional review board approved the study, and all parents of the infant patients provided informed consent for study inclusion (Date: 30 December 2021).

Inclusion criteria: (1) infants under 1 year old; (2) Clinical manifestations: developmental delay; bulging fontanel, high tension; ever; (3) Cranial CT results suggest SE thickness ≥ 1.5 cm; (4) Cerebrospinal fluid (CSF) results suggest abnormal white blood cells, protein, and sugar; (5) Accept high-pressure oxygen and/or subdural drilling and drainage; (6) Parents sign informed consent for surgery and postoperative follow-up; (7) No other diseases, can tolerate surgery or high-pressure oxygen therapy.

Exclusion criteria: (1) Children over 1 year old; (2) Cranial CT results indicating SE thickness < 1.5 cm; (3) Trauma, intracranial tumor surgery, and other non-infectious factors causing SE; (4) Genetic metabolic diseases; (5) Previous history of craniotomy surgery; (6) Abandoning treatment or death halfway; (7) Inability to tolerate surgery due to disease.

**Figure 1:**
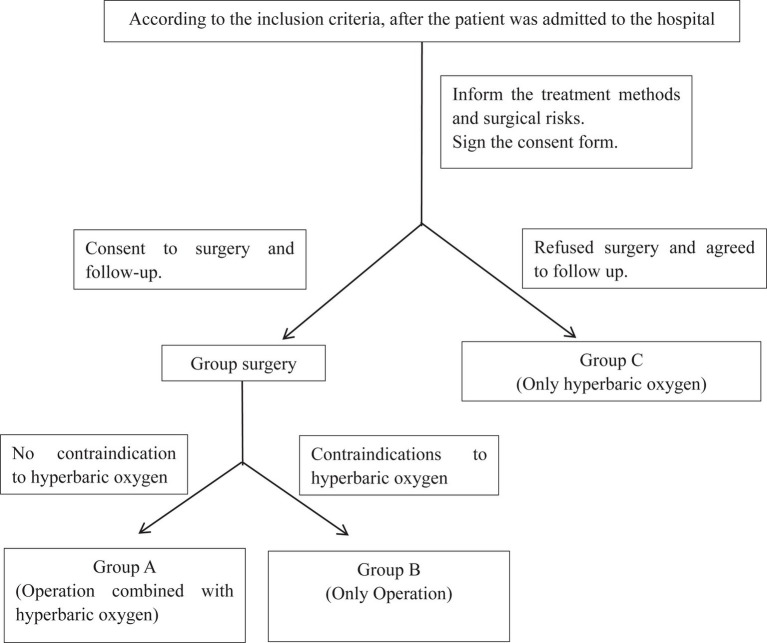
Grouping flow chart.

Grouping principle: Inform parents of the surgical risks of SDD, and perform surgical treatment after parents understand and sign the informed consent form. After the surgical incision heals, perform physical assessment before hyperbaric oxygen therapy for children; eyeground hemorrhage, otitis media, etc. are not suitable for hyperbaric oxygen therapy. Children with normal physical examination are in group A, those with hyperbaric oxygen contraindications are in group B. Children whose parents believe that surgery is risky and refuse surgical treatment belong to group C (Figure 1).

According to the inclusion and exclusion criteria, a total of 328 infant patients of SE type 4 with intracranial infection were included in this study. Among them, 178 infant patients were treated by hyperbaric oxygen combined with SDD (group A), including 101 boys and 77 girls, with an average age of 4.6 ± 0.84 months. 142 cases (group B) were treated with SDD, including 78 boys and 64 girls, with an average age of 4.5 ± 0.62 months. 97 children (group C) were treated with hyperbaric oxygen only, including 37 boys and 60 girls, with an average age of 4.2 ± 0.73 months.

The treatment process of hyperbaric oxygen usually includes preparation before pressurization, pressurization, pressure stabilization, decompression, and observation of the patient’s condition after decompression. The hyperbaric oxygen chambers in this study are all single-person chambers. Before pressurization, the doctor will adjust the parameters of the hyperbaric oxygen chamber based on the condition, age, and weight of the patient. The children in this study were similar in age and weight, and the pressure setting was 1.6ATA. Because the child was young, he/she would usually be accompanied by a parent. The parent would also have his eyes and ears examined before entering the hyperbaric oxygen chamber. Parents and children kept comfortable posture after entering the hyperbaric oxygen chamber. The doctor will inform the parents to start pressurization. The hyperbaric oxygen chamber is equipped with a microphone. If parents or children experience any discomfort, they can communicate with the doctor through the microphone. There is a transparent window in the front of the hyperbaric oxygen chamber, so the doctor can observe the state of the child. The pressure-maintaining treatment lasts for 30 min. After the treatment is completed, the pressure is reduced. The doctor will inform the parents to start decompression and observe the patient’s condition through the window. After leaving the chamber, the doctor will examine the parents and child and observe the condition in the ward to avoid decompression sickness. If the child has abnormal crying during the treatment, the treatment will be stopped immediately and a screening of the fundus and ears will be conducted. Hyperbaric oxygen therapy is administered once a day for 10 consecutive days as a course of treatment. A course of treatment is carried out every month.

### Clinical evaluation

According to the measurement of the maximum thickness of the subdural residual cavity using head CT, the degree of SE is divided into four types: Type I: T < 0.5 cm. Type II: 0.5 cm ≤ T < 1 cm. Type III: 1 cm ≤ T < 1.5 cm. Type IV T ≥ 1.5 cm. Compare the degree of brain re-expansion between the three groups of children before and 6 months after treatment. Record the results of CSF culture and the time of intracranial infection control. Evaluate the prognosis of the patients. Record the complications.

### Operation procedures

**Figure 2:**
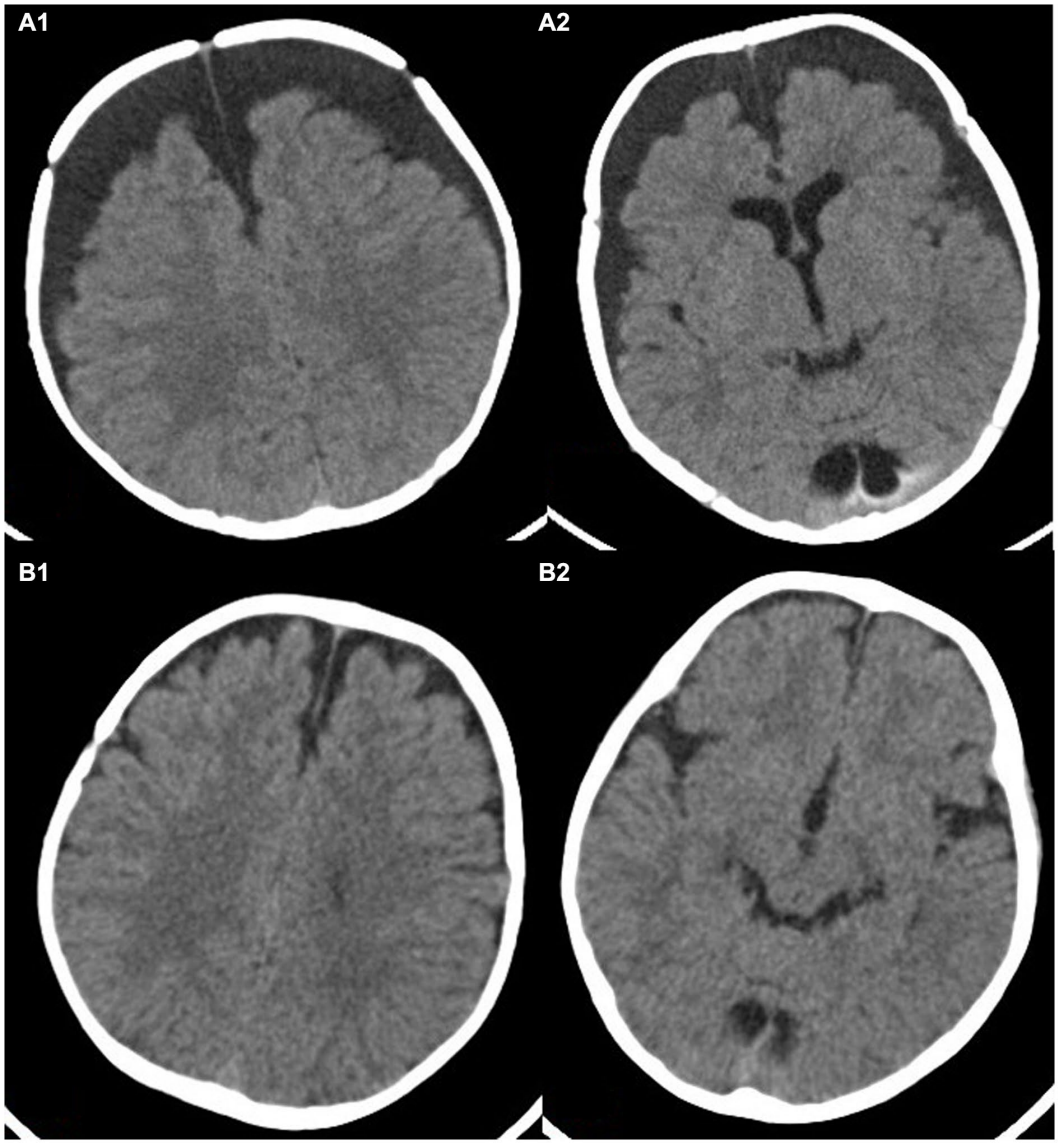
This image shows a 4-month-old child with subdural effusion in bilateral frontotemporal parietal lobe in **(A1,A2)**, with a subdural residual cavity thickness greater than 1.5 cm. Brain tissue atrophy is also visible. Panels **(B1,B2)** show the extent of brain re-expansion after 6 months of hyperbaric oxygen therapy. The bilateral subdural residual cavities have significantly improved.

**Figure 3:**
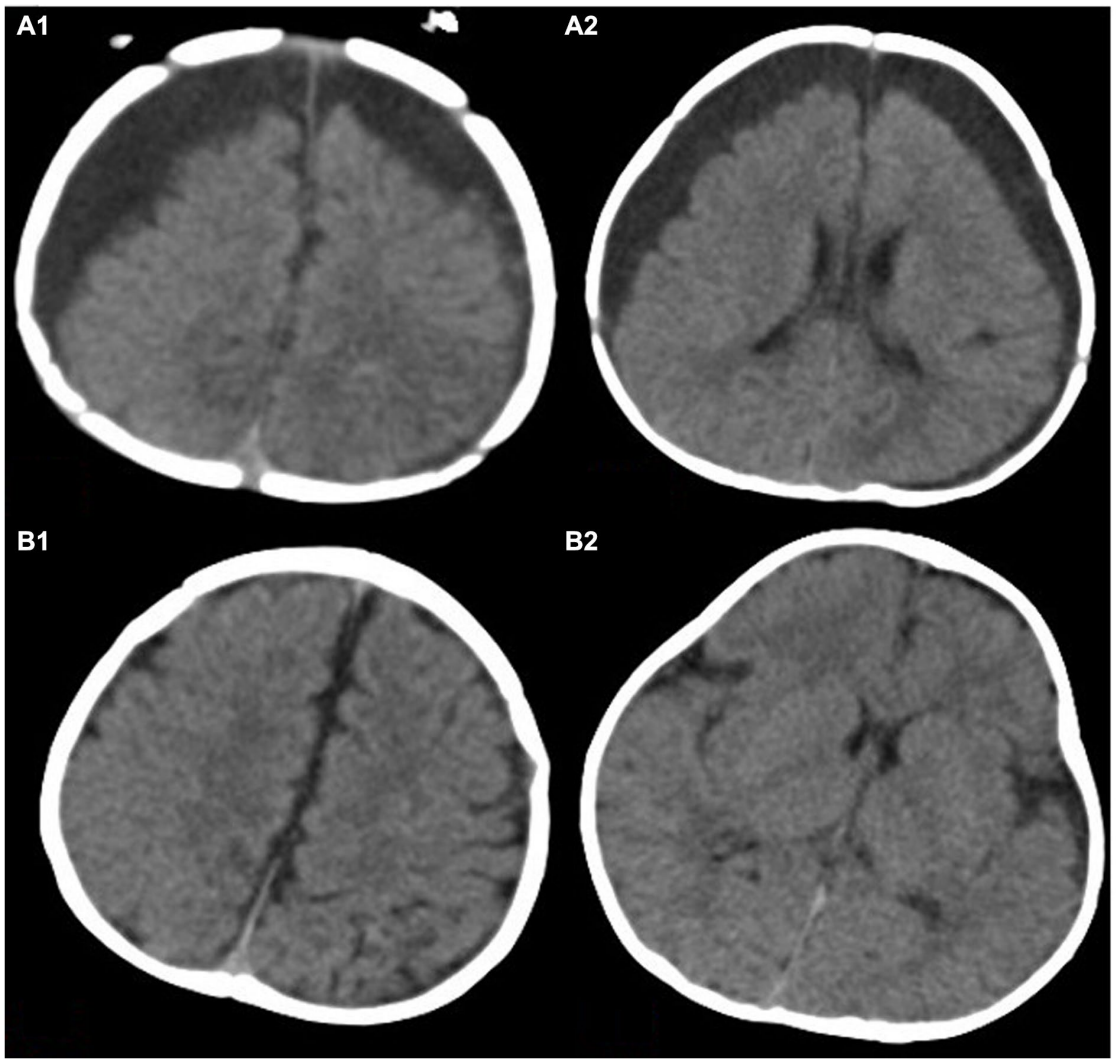
This image shows a 3.5-month-old child with bilateral subdural effusion in frontotemporal parietal lobe in **(A1,A2)**. The thickness of subdural residual cavity at the widest part is more than 2.5 cm. The brain tissue is obviously atrophied. Panels **(B1,B2)** show the degree of brain re-expansion after hyperbaric oxygen therapy. The brain tissue is visibly full.

According to the CT scan of the skull, locate the surgical incision at the thickest point of subdural fluid accumulation. Make a straight incision of approximately 2.0 cm in length and cut the skin in sequence. Drill a hole in the skull with a diameter of about 0.7 cm, cut the dura mater in a T-shaped incision, and yellow CSF can be seen overflowing. Reserve a portion of the CSF for examination. Place a silicone tube into the subdural cavity about 2-4 cm, and repeatedly rinse with vancomycin saline until the flushing fluid is colorless and transparent. Thread the drainage tube through a small incision on the scalp, fix the drainage tube, intermittently suture the skin, and connect the distal end to a closed drainage device. Use the same surgical method for contralateral subdural effusion (Figures 2, 3).

### Postoperative treatment

On the first day after surgery, recheck the head CT to understand the position of the drainage tube. The drainage device limits the drainage fluid to 100 mL per day. The drainage flow can be adjusted according to symptoms. The type of antibiotic used is determined based on the results of CSF culture, and sensitive antibiotics are injected intrathecally. Based on the characteristics of the drainage fluid, the extent of brain re-expansion is removed from the head drainage tube. Generally, the drainage tube is left in place for 7–10 days. CSF is rechecked every 3 days. Normal results of two CSF tests are considered to indicate that intracranial infection has been controlled. Intermittent head CT or MRI is performed to check the brain re-expansion of the infant patients. After removing the drainage tube, hyperbaric oxygen therapy is started.

### Statistical analysis

Statistical analysis was performed using SPSS 26.0 (IBM, Armonk, New York, United States) software. Normal distribution of quantitative data was described by mean ± standard deviation (^−^X ± S), and non-normal distribution of quantitative data was described by median. The pre- and post-treatment data (CSF status, brain re-expansion degree, etc.) in each group were analyzed using t-tests, and the data of multiple groups (intracranial infection control time, degree of brain re-expansion.) were compared using F-tests. Count data were expressed as number and percentage (n, %), and the difference between the two groups was evaluated using X^2^ test or Fisher’s exact test. Statistical significance was considered when *p* < 0.05.

## Results

After treatment, the clinical symptoms and various examination indicators of the three groups of patients were significantly improved. The average cure time for intracranial infection in the hyperbaric oxygen combined with SDD group (Group A) was 18.2 ± 5.9 days. The average time for controlling intracranial infection in the SDD group (Group B) was 19.1 ± 11.2 days. The average time for controlling intracranial infection in the hyperbaric oxygen group (Group C) was 23.2 ± 7.5 days. There was no statistically significant difference between Group A and Group B (*p* < 0.05), while Group A and Group B were statistically significant compared to Group C (*p* > 0.05).

At a 6-month follow-up, the degree of brain re-expansion in the three groups of patients was significantly improved compared to before treatment, with a statistically significant difference. Before treatment, the thickness of the subdural residual cavity in Group A was 2.3 ± 0.61 cm, and after surgery, it was 0.6 ± 0.50 cm. The thickness of group B before drilling was 2.2 ± 0.46 cm, and the thickness of the residual cavity after treatment was 0.8 ± 0.49 cm. Group C is a hyperbaric oxygen group, with a residual cavity thickness of 2.1 ± 0.58 cm before treatment and 0.8 ± 0.51 cm after treatment. There was no statistically significant difference in the thickness of the subdural residual cavity before treatment among the three groups of patients. The degree of brain re-expansion after treatment was significantly improved compared to before treatment, with a statistically significant difference. It is worth noting that after treatment, there was a significant difference between Group A and Group B, as well as Group C, with statistical significance (*p* < 0.05). There was no significant difference in the degree of brain re-expansion between Group B and Group C, and there was no statistically significant difference (*p* > 0.05; Table 1).

**Table 1:** Comparison of brain re-expansion in three groups of infant patients before and after treatment.

	Group A	Group B	Group C	*F*	*P* ^a^	*P* ^b^	*P* ^c^
Before treatment	2.3 ± 0.61	2.2 ± 0.46	2.1 ± 0.58	2.74	>0.05	>0.05	>0.05
After 6 months of treatment	0.6 ± 0.50	0.8 ± 0.49	0.8 ± 0.51	9.61	<0.01	<0.01	>0.05
t	27.84	25.39	16.82				
*P*	0.000	0.000	0.000				

*P^a^* is the comparison between group A and group B. *P^b^* is the comparison between group A and group C. *P^c^* is the comparison between group B and group C.

There were 96 cases of type 1 (53.9%), 53 cases of type 2 (29.8%), 22 cases of type 3 (12.4%), and 7 cases of type 4 (3.9%) in group A. The degree of brain recruitment in Group B was 46 cases (32.4%) of Type 1, 53 cases (29.8%) of Type 2, 22 cases (12.4%) of Type 3, and 12 cases (8.5%) of Type 4, respectively. The degree of brain recruitment in Group C was 30 cases (30.9%) of Type 1, 25 cases (25.8%) of Type 2, 35 cases (36.1%) of Type 3, and 7 cases (7.2%) of Type 4, respectively. Based on the comprehensive evaluation of the treatment effect and imaging results, the treatment effect is divided into four levels: cured, significantly improved, improved, and ineffective. The sum of cure rate and significant effect is the effective rate. The effective rate of Group A was 83.7%, Group B was 58.5%, and Group C was 56.7% (Table 2).

**Table 2:** Comparison of the effective rate and grading of the three groups of patients after treatment.

	Type I-cured (%)	Type II-significantly Effective (%)	Type III-improved (%)	Type IV-ineffective (%)	Effective rate (%)
Group A	96 (53.9%)	53 (29.8%)	22 (12.4%)	7 (3.9%)	149 (83.7%)
Group B	46 (32.4%)	37 (26.1%)	47 (33.1%)	12 (8.5%)	83 (58.5%)
Group C	30 (30.9%)	25 (25.8%)	35 (36.1%)	7 (7.2%)	55 (56.7%)
*X* ^2^					32.1
*P*					0.000

Effective rate = Cure rate + Significantly Effective.

There were 22 patients with subcutaneous fluid accumulation after surgery in group A, and 22 patients in group B. Both groups showed improvement in subcutaneous fluid accumulation after pressure dressing of the surgical incision. No adverse symptoms occurred in group C.

## Discussion

SE is often caused by trauma, purulent meningitis. It is common in frontotemporal lobe or frontotemporal parietal lobe, and most subdural effusion is bilateral (2). The cause of traumatic SE is generally believed to be the movement of brain tissue within the cranial cavity during head trauma, resulting in tearing of the lateral fissure cistern, optic chiasma cistern, and arachnoid membrane on the brain surface. CSF flows from the perforation to the subdural space between the subdural and arachnoid membranes and accumulates. The main mechanism is that the perforation of the arachnoid membrane forms a unidirectional valve that cannot flow back (1, 13, 14). Babies are prone to crying. The forceful movement during crying also accelerates the outflow of CSF to some extent, ultimately forming SE. As the accumulation of fluid increases, brain tissue compression becomes apparent, and intracranial pressure gradually increases.

Purulent meningitis with SE is common in infants and young children. The pathogenesis is inflammation and embolism in the dura mater, bridging veins, and superficial veins of cerebral blood vessels. Exudation and bleeding increase local osmotic pressure, causing surrounding water to flow into the subdural cavity, resulting in subdural effusion (2, 15). Another reason is the increased permeability of brain tissue and meningeal vascular walls, and the entry of plasma albumin into the subdural cavity to form SE. After the formation of SE, it can lead to compression of brain tissue, gradual atrophy of brain tissue, and even permanent neurological sequelae in some children (6). Patients with some SE may also experience subdural bleeding (16). The main reason is that long-term accumulation of fluid under the dura mater can form a capsule, which gradually increases, leading to bridging vein rupture and bleeding or capsule bleeding. Excessive fibrinolysis in the submucosal fluid can also lead to abnormal coagulation function, exacerbating bleeding (17, 18). There is another reason that has not been clearly confirmed. When the children cry, some parents hold them in their arms and toss them up and down to seek to soothe their emotions. When the number of bumps is high or the amplitude of movements is large, it may cause slight displacement of brain tissue, leading to rupture and bleeding of bridging veins or other blood vessels. Intracranial infection accompanied by subdural fluid and blood accumulation may lead to severe disruption of the internal environment of brain tissue. The situation of brain atrophy will gradually worsen, and the likelihood of brain re-expansion in patients will be lower. It will seriously affect the prognosis, gradually leading to sequelae of the nervous system, and even cerebral palsy. Therefore, active and effective treatment measures should be taken for children with SE.

The current treatment methods for SE include anterior fontanelle puncture, venous indwelling needle drainage, drilling drainage, subdural peritoneal shunt, and craniotomy. The advantage of anterior fontanel puncture is that it is easy to operate and can be performed on the patient’s bed. However, repeated puncture can easily cause subdural hemorrhage and iatrogenic intracranial infection, and repeated puncture can bring certain pain to the patient (10). Although venous indwelling needle drainage avoids repeated puncture and reduces the possibility of subdural bleeding. However, bilateral subdural fluid accumulation and placement of a tube at the anterior fontanel can cause the position of the anterior fontanel to be particularly prominent and not firmly fixed (2). Subdural intraperitoneal shunt should be avoided in cases of intracranial infection, as the shunt tube may become blocked during infection, leading to surgical failure (11). The indications for craniotomy surgery mainly include refractory SE, the formation of encapsulated cystic wall, and the attachment of the cystic wall to the surface of the brain tissue, completely limiting the development of the child’s brain tissue (12).

SDD is a minimally invasive surgical procedure. This surgical approach has a small incision, simple, and minimal trauma to the infant. During the operation, warm saline is used to repeatedly flush the subdural cavity until the effusion color is colorless and transparent. By replacing the SE, the internal environment of the brain tissue can be improved. The drainage tube is a soft tube that does not affect the movement of the child’s head when fixed on the head. After surgery, dynamic detection of CSF is performed to timely understand the condition of intracranial infection, so as to dynamically adjust the treatment plan. SDD can drain the exudate and inflammatory factors of a large number of pathogenic bacteria in the SE, reduce the bacterial concentration in the effusion (5). At the same time, it also accelerates the circulation of SE, improves the internal environment of brain tissue, reduces intracranial pressure, and alleviates the symptoms of brain atrophy. After surgery, antibiotics can be injected through the drainage tube, crossing the blood–brain barrier, and the drug rapidly diffuses on the brain surface, effectively reaching the drug treatment concentration in the infected area, thus effectively controlling the infection and improving the prognosis of the children (19–21). In this study, all children were treated with empirical anti-infective therapy with cefotaxime and sulbactam sodium until the etiology results were clear. After surgery, they were treated with empirical anti-infective therapy with intrathecal vancomycin. After the etiology results were announced, the most sensitive antibiotic was used for intrathecal anti-infective therapy.

A total of 320 children (group A and B) in this study underwent SDD. Through dynamic monitoring of the effusion results after surgery, it was found that the white blood cell count, protein, and glucose content in the SE decreased significantly by day 7 after surgery. The color of the drainage fluid also gradually became lighter. The fever symptoms improved significantly. The condition was basically stable by 2 weeks after surgery. Sun (22) believes that after controlling intracranial infection, the inflammatory mediators in SE will gradually decrease. The inflammation of the dura mater, bridging veins, and superficial cerebral veins will also gradually alleviate, and the venous circulation will be unobstructed. As the inflammation is controlled, the vascular permeability on the surface of the brain gradually recovers, with no protein leakage, and the local osmotic pressure gradually returns to normal. Eventually, the SE is gradually controlled (23). In this study, the time to control intracranial infection in group A was 18.2 ± 5.9 days, and in group B it was 19.1 ± 11.2 days, both of which were better than the time to control infection in group C (23.2 ± 7.5 days). This further confirms the effectiveness of drilling and drainage combined with intrathecal drug injection in the treatment of SE with infection. A small number of children in group A and B had longer treatment times than some children in group C, which we believe may be related to the severity of purulent meningitis or the infection of specific bacteria. Even with aggressive treatment, the time for infection control will be slightly longer. However, the changes in individual samples will not affect the overall cure rate.

Hyperbaric oxygen therapy means that the body absorbs oxygen in an environment with higher than atmospheric pressure (8). Hyperbaric oxygen has a positive effect on the recovery of various diseases such as ischemic and hypoxic encephalopathy, cranial brain injury, and spinal cord injury (24). Hyperbaric oxygen therapy can promote vascular dilation in ischemic brain tissue, which can increase the oxygen content and concentration in the diseased brain tissue (25). Thus, it promotes the aerobic metabolism of brain tissue and the repair of nerve cells. The volume of the cerebral gyrus gradually increases, the SE gradually absorbs, and the subdural space gradually shrinks. At the same time, hyperbaric oxygen can also promote the regeneration of capillary vessels in the lesion area, promote the establishment of collateral circulation, and have a significant positive effect on the establishment of cerebral vascular microcirculation (26, 27). On the other hand, hyperbaric oxygen can also significantly improve the microcirculation system of brain tissue, promote the recovery of brain tissue, and accelerate the absorption of SE (28). Some literature suggests that hyperbaric oxygen therapy can improve the permeability of the cerebral vascular wall and blood–brain barrier, and accelerate the absorption of SE (24, 29). Hadanny et al. believed that hyperbaric oxygen therapy not only improved the permeability of the blood–brain barrier, but also facilitated the passage of neurotrophic drugs through the blood–brain barrier into the brain tissue, which had a repairing effect on brain atrophy and brain injury (30).

In this study, after 6 months of treatment, the subdural residual cavity of patients in group A increased from 2.3 ± 0.61 cm before treatment to 0.6 ± 0.50 cm after treatment. Among them, 96 patients had a subdural residual cavity thickness of 0.3 ± 0.11 cm. 53 patients had a subdural residual cavity thickness of 0.7 ± 0.16 cm. The effective rate was 83.7%. At the last follow-up, the subdural residual cavity of patients in both group B and group C improved to some extent, with the effective rates of 58.5 and 56.7%, respectively. However, compared with group A, the effective rate of group A was significantly higher than the other two groups, with significant statistical differences. The research results indicate that hyperbaric oxygen therapy has a positive effect on promoting the expansion of brain tissue in children with cerebral palsy. Patients in group B did not receive hyperbaric oxygen therapy due to otitis media or fundus hemorrhage. Although the time of intracranial infection was statistically significant compared to group C, there was no significant difference in the degree of brain re-expansion and efficacy compared to group C. This result suggests that timely control of infection also has a positive effect on brain retraction. However, the parents of children in group C refused surgical treatment considering the risk of surgery. The time required to control intracranial infection was slightly longer, but after active hyperbaric oxygen therapy, the degree of brain re-expansion was similar to that in group B. This result also reaffirms the role of hyperbaric oxygen therapy.

This study suffered from several limitations. First, the retrospective design might have impeded the accuracy and precise in data collection. Second, due to the limited use in our institution, only 328 eligible patients were included for data analysis, making the comparison not definitely conclusive. Third, the single-center design would have lowered the generalizability of our results to other settings.

## Conclusion

The SDD is safe and effective for infant patients with intracranial infections through fluid replacement and intrathecal antibacterial. Hyperbaric oxygen is effective as an adjuvant therapy to promote brain re-expansion. This treatment method has achieved good clinical results. Future research should further explore individualized treatment for patients, so as to establish a unified and standardized diagnostic and treatment strategy, making it more clinically instructive.

## Data availability statement

The original contributions presented in the study are included in the article/supplementary material, further inquiries can be directed to the corresponding author.

## Ethics statement

The studies involving human participants were reviewed and approved by the Institutional Review Board of Hebei Children’s Hospital (Approval number: 0220735). Written informed consent to participate in this study was provided by the patients/participants or patients/participants’ legal guardian/next of kin.

## Author contributions

LC: Data curation, Methodology, Writing – original draft, Formal analysis, Software. YY: Data curation, Formal analysis, Software, Writing – original draft, Resources. PL: Data curation, Software, Writing – original draft, Validation. YQ: Formal analysis, Methodology, Resources, Writing – original draft. JF: Methodology, Investigation, Software, Writing – original draft. CX: Investigation, Resources, Writing – original draft. LL: Resources, Data curation, Writing – original draft. JL: Data curation, Investigation, Writing – original draft. ZC: Resources, Supervision, Validation, Visualization, Writing – original draft. ZY: Supervision, Visualization, Project administration, Writing – review & editing. YS: Supervision, Conceptualization, Data curation, Investigation, Methodology, Resources, Writing – original draft, Writing – review & editing.

## References

[1] Jian-Yun Z Xin Z Hai-Bin G Cao Z Sun W. Endoscopic-assisted surgery for skull defects with subdural effusion. Wideochir Inne Tech Maloinwazyjne. (2021) 16:219–26. doi: 10.5114/wiitm.2020.99350 33786137 PMC7991952

[2] Wang X Zhang X Cao H Jing S Yang Z Cheng Z. Surgical treatments for infantile purulent meningitis complicated by subdural effusion. Med Sci Monit. (2015) 21:3166–71. doi: 10.12659/MSM.895747, PMID: 26482715 PMC4621163

[3] Ban SP Son YJ Yang HJ Chung YS Lee SH Han DH. Analysis of complications following decompressive craniectomy for traumatic brain injury [J]. Korean Neurosurg Soc. (2010) 48:244–50. doi: 10.3340/jkns.2010.48.3.244, PMID: 21082053 PMC2966727

[4] Xu YJ Wang Q Su L Dai XY Zhu XY. Analysis of scores of SCL-90 of patients with traumatic subdural effusion. Fa Yi Xue Za Zhi. (2020) 36:223–8. doi: 10.12116/j.issn.1004-5619.2020.02.014, PMID: 32530171

[5] Chen L He M Shi L Yue Y Luo P Fang J. Effects of modified external ventricular drainage vs. an Ommaya reservoir in the management of hydrocephalus with intracranial infection in pediatric patients. Front Neurol. (2024) 14:1303631. doi: 10.3389/fneur.2023.1303631, PMID: 38274873 PMC10808584

[6] Namani SA Koci RA Kuchar E Dedushi KH. Surgical treatment of neurologic complications of bacterial meningitis in children in Kosovo. J Trop Pediatr. (2012) 58:139–42. doi: 10.1093/tropej/fmr040, PMID: 21873279

[7] Vasilopoulou VA Karanika M Theodoridou K Katsioulis AT Theodoridou MN Hadjichristodoulou CS. Prognostic factors relat-ed to sequelae in childhood bacterial meningitis: data from a Greek men-ingitis registry. BMC Infect Dis. (2011) 11:214. doi: 10.1186/1471-2334-11-214, PMID: 21827712 PMC3166933

[8] Aydin F. Hyperbaric oxygen treatment in children: experience in 329 patients[J]. Diving Hyperb Med. (2023) 53:203–9. doi: 10.28920/dhm53.3.203-209, PMID: 37718293 PMC10735713

[9] Laureau J Pons C Letellier G Gross R. Hyperbaric oxygen in children with cerebral palsy: a systematic review of effectiveness and safety. PLoS One. (2022) 17:e0276126. doi: 10.1371/journal.pone.0276126, PMID: 36240157 PMC9565562

[10] Wan S Dong W. A case of bilateral subdural effusion treated with puncture inside angle of anterior Fontanelle. Chin J Pediatr. (2000) 38:51. doi: CNKI:SUN:ZHEK.0.2000-06-021

[11] Guo H Zhou X Li X Yang S Wang Y. Scenario for the use of effusion-peritoneal shunt necessary against subdural effusion secondary to decompressive craniectomy. Clin Neurol Neurosurg. (2021) 203:106598. doi: 10.1016/j.clineuro.2021.106598, PMID: 33730617

[12] Klimo PJ Matthews A Lew SM Zwienenberg-Lee M Kaufman BA. Minicraniotomy versus bur holes for evacuation of chronic subdural collections in infants-a preliminary single-institution experience. J Neurosurg Pediatr. (2011) 8:423–9. doi: 10.3171/2011.8.PEDS1131, PMID: 22044363

[13] Liu Y Gong J Li F Wang H Zhu S Wu C. Traumatic subdural hydroma: clinical characteristics and classification. Injury. (2009) 40:968–72. doi: 10.1016/j.injury.2009.01.006 19540485

[14] Yaodong W Chuanwei W Yuguang L. Chronic subdural haematoma evolving from traumatic subdural hydroma. Brain Inj. (2015) 29:462–5. doi: 10.3109/02699052.2014.990513 25536390

[15] Imataka G Miyamoto K Fujiyama Y Mitsui M Yoshida A Yamanouchi H. Acute purulent meningitis associated with chronic subdural hematoma and subdural hygroma. Turk J Pediatr. (2007) 49:437–40. PMID: 18246750

[16] Tao Z Lin Y Hu M Ding S Li J Qiu Y. Mechanism of subdural effusion evolves into chronic subdural hematoma: IL-8 inducing neutrophil oxidative burst. Med Hypotheses. (2016) 86:43–6. doi: 10.1016/j.mehy.2015.11.027 26804595

[17] Wan Y Shi L Wang Z Sun G Pan T Zhang S. Effective treatment via early cranioplasty for intractable contralateral subdural effusion after standard decompressive craniectomy in patients with severe traumatic brain injury. Clin Neurol Neurosurg. (2016) 149:87–93. doi: 10.1016/j.clineuro.2016.08.004 27500656

[18] Wang Z-f Liao D-g. Role of matrix metalloproteinase in ransformation of subdural effusion into chronic subdural hematoma. Nan Fang Yi Ke Da Xue Xue Bao. (2010) 30:1188–9. doi: 10.12122/j.issn.1673-4254.2010.05.074 PMID: 20501425

[19] Sullins AK Abdel-Rahman SM. Pharmacokinetics of antibacterial agents in the CSF of children and adolescents[J]. Paediatr Drugs. (2013) 15:93–117. doi: 10.1007/s40272-013-0017-5, PMID: 23529866

[20] Chen FM Deng X Wang Z Wang L Wang K Gao L. Treatment of severe ventriculitis caused by extensively drug-resistant *Acinetobacter baumannii* by intraventricular lavage and administration of colistin [J]. Infect Drug Resist. (2019) 12:241–7. doi: 10.2147/IDR.S186646, PMID: 30718963 PMC6345184

[21] Nau R Blei C Eiffert H. Intrathecal antibacterial and antifungal therapies. Review Clin Microbiol Rev. (2020) 33:e00190–19. doi: 10.1128/CMR.00190-19, PMID: 32349999 PMC7194852

[22] Sun TFD Boet R Poon WS. Non-surgical primary treatment of chronic subdural haematoma: preliminary results of using dexamethasone. Br J Neurosurg. (2005) 19:327–33. doi: 10.1080/02688690500305332 16455539

[23] Okada Y Akai T Okamoto K Iida T Takata H Iizuka H. A comparative study of the treatment of chronic subdural hematomaburr hole drainage versus burr hole irrigation[J]. Surg Neurol. (2002) 57:405–9. doi: 10.1016/S0090-3019(02)00720-6, PMID: 12176202

[24] Ortega MA Fraile-Martinez O García-Montero C Callejón-Peláez E Sáez MA Álvarez-Mon MA. A general overview on the hyperbaric oxygen therapy: applications, mechanisms and translational opportunities. Medicina. (2021) 57:864:864. doi: 10.3390/medicina57090864, PMID: 34577787 PMC8465921

[25] Muir ER Cardenas DP Duong TQ. MRI of brain tissue oxygen tension under hyperbaric conditions. NeuroImage. (2016) 133:498–503. doi: 10.1016/j.neuroimage.2016.03.040, PMID: 27033683 PMC5313390

[26] Xue L Yu Q Zhang H Liu Y Wang C Wang Y. Effect of large dose hyperbaric oxygenation therapy on prognosis and oxidative stress of acute permanent cerebral ischemic stroke in rats. Neurol Res. (2008) 30:389–93. doi: 10.1179/174313208X300413, PMID: 18544257

[27] Shi-yuan J Xian-shu W Zhi-guo Y Zheng-hai C Hong-bin C Xin L. The therapeutic effect of hyperbaric oxygen on the postoperative brain re-expansion in children with in tractable subdura leffusion. Chin J Pediatr Surg. (2013) 34:919–21. doi: 10.3760/cma.j.issn.0253-3006.2013.12.012

[28] Tal S Hadanny A Sasson E Suzin G Efrati S. Hyperbaric oxygen therapy can induce angiogenesis and regeneration of nerve fibers in traumatic brain injury patients. Front Hum Neurosci. (2017) 11:508. doi: 10.3389/fnhum.2017.00508, PMID: 29097988 PMC5654341

[29] Rockswold SB Rockswold GL Defillo A. Hyperbaric oxygen in traumatic brain injury. Neurol Res. (2007) 29:162–72. doi: 10.1179/016164107X181798 17439701

[30] Hadanny A Golan H Fishlev G Bechor Y Volkov O Suzin G. Hyperbaric oxygen can induce neuroplasticity and improve cognitive functions of patients suffering from anoxic brain damage. Restor Neurol Neurosci. (2015) 33:471–86. doi: 10.3233/RNN-150517, PMID: 26409406 PMC4923708

